# Supplementary figures and images for: Critical roles for ‘housekeeping’ nucleases in type III CRISPR-Cas immunity
Source: eLife. 2022 Dec 8;11:e81897. doi: 10.7554/eLife.81897 (PMC9762709; doi:10.7554/eLife.81897)

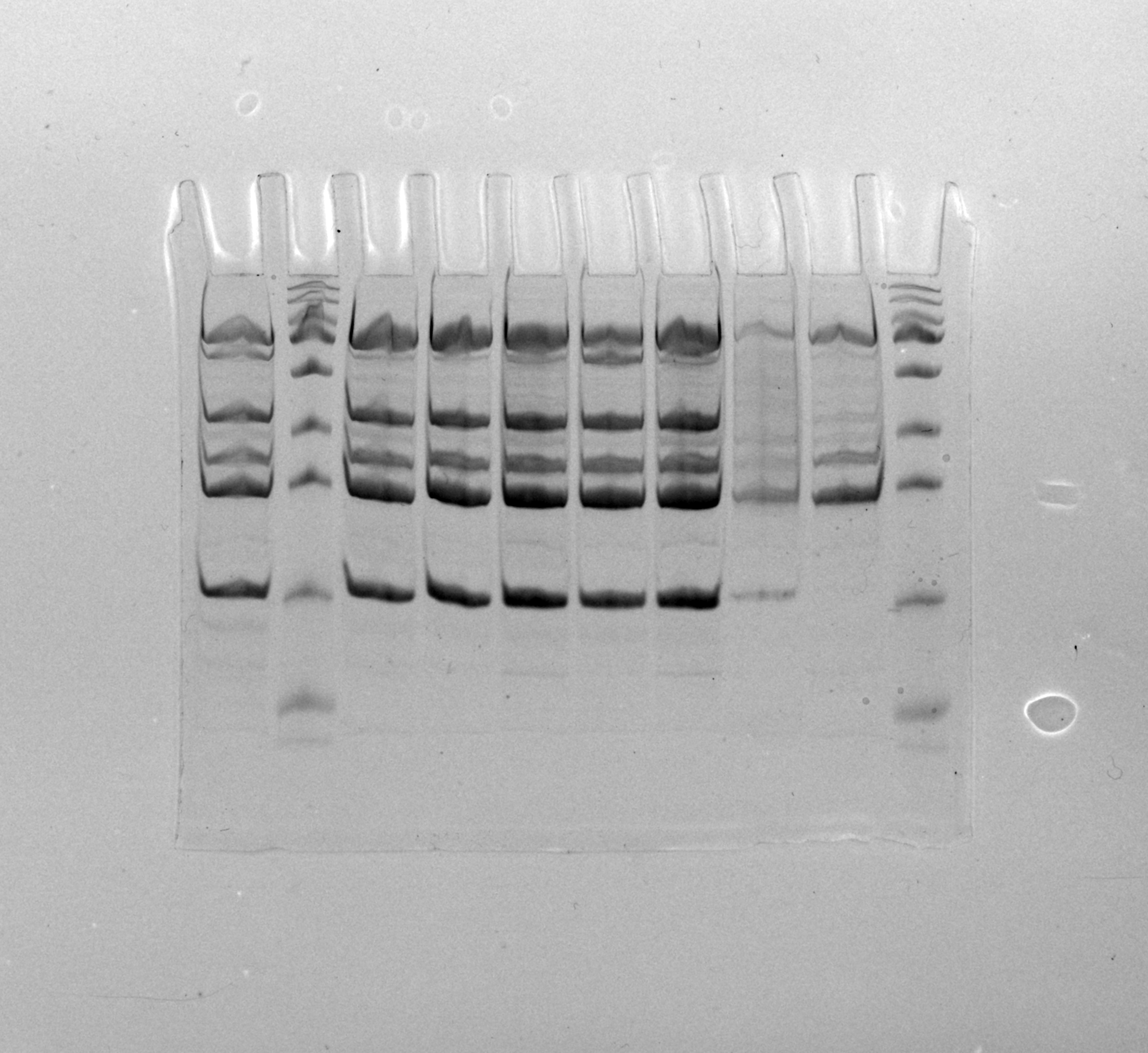

Supplement: Figure 1—source data 1. [file elife-81897-fig1-data1.zip › Figure 1-source data 1/Figure 1D_raw_uncropped.tif]

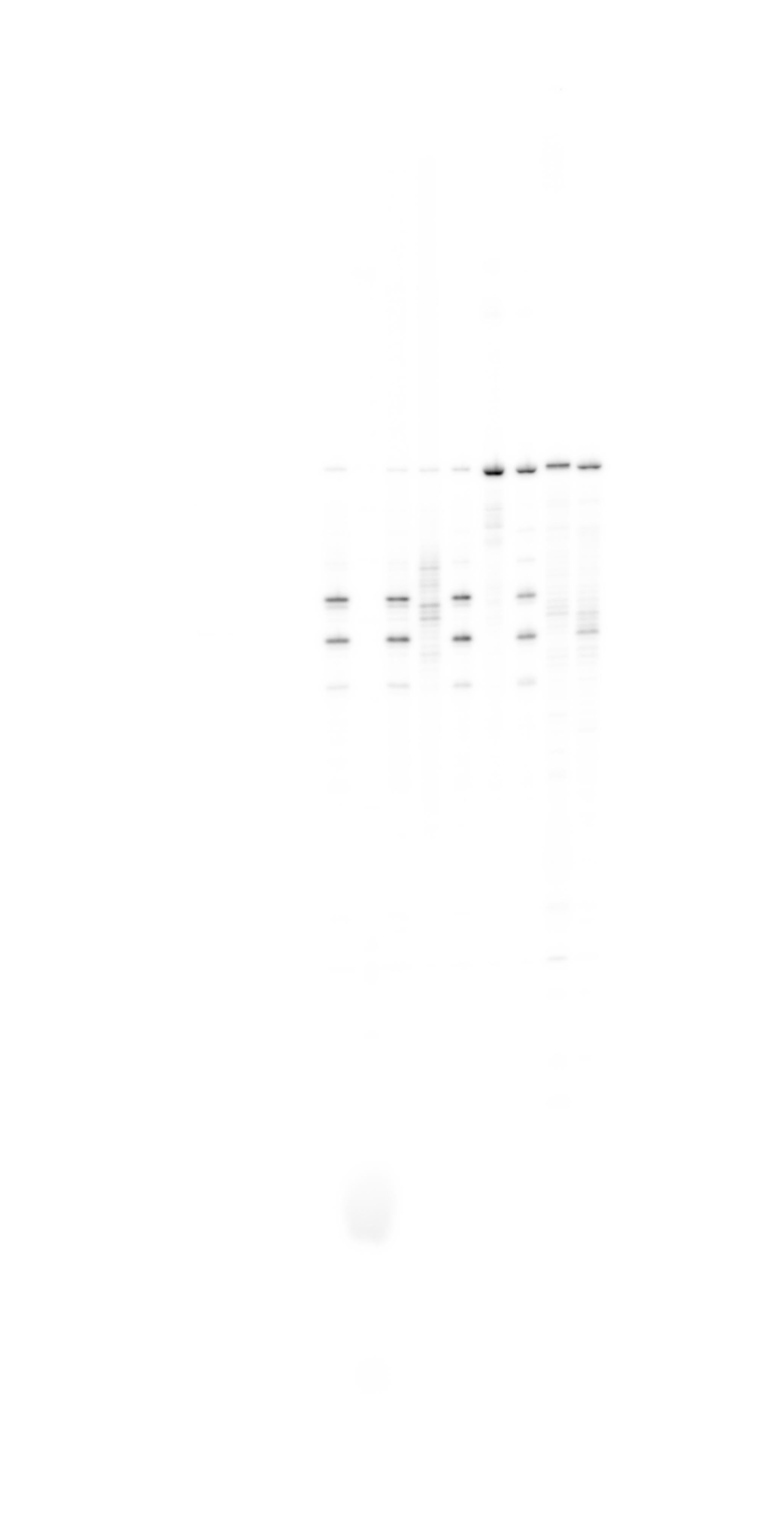

Supplement: Figure 1—source data 2. [file elife-81897-fig1-data2.zip › Figure 1-source data 2/Figure 1E_raw_uncropped.tif]

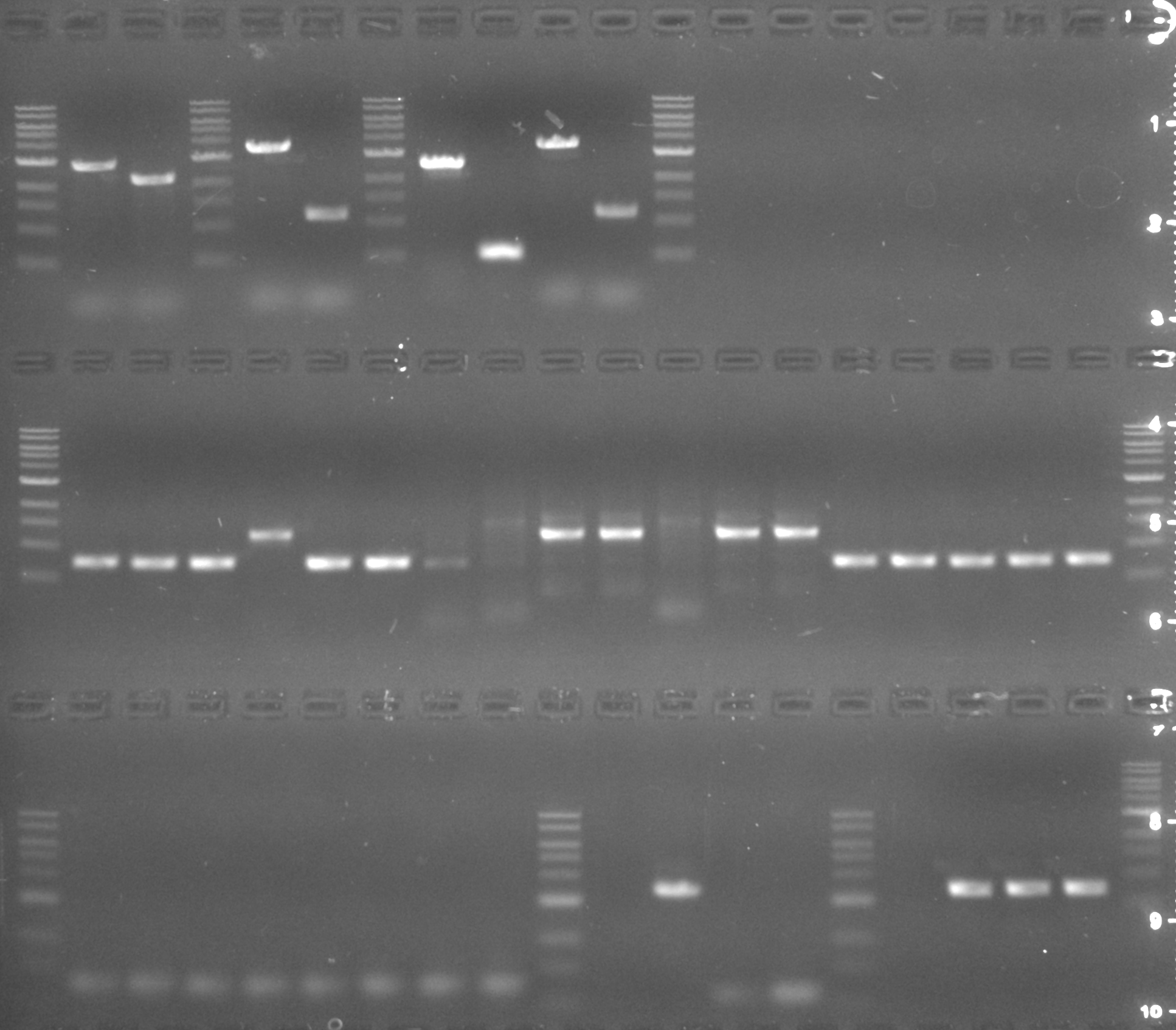

Supplement: Figure 1—figure supplement 1—source data 1. [file elife-81897-fig1-figsupp1-data1.zip › Figure 1-figure supplement 1-source data 1/Figure 1-figure supplement 1-source data 1_raw_uncropped.tif]

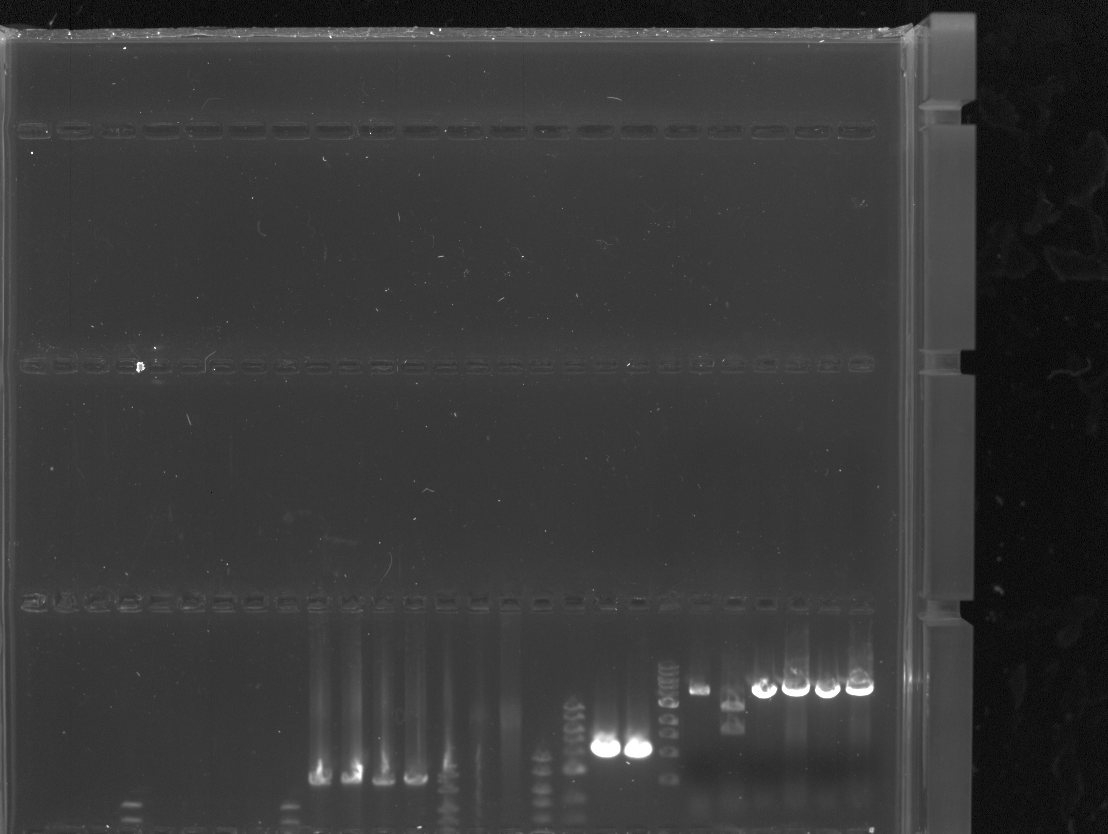

Supplement: Figure 1—figure supplement 1—source data 2. [file elife-81897-fig1-figsupp1-data2.zip › Figure 1-figure supplement 1-source data 2/Figure 1-figure supplement 1-source data 2-raw_uncropped.png]

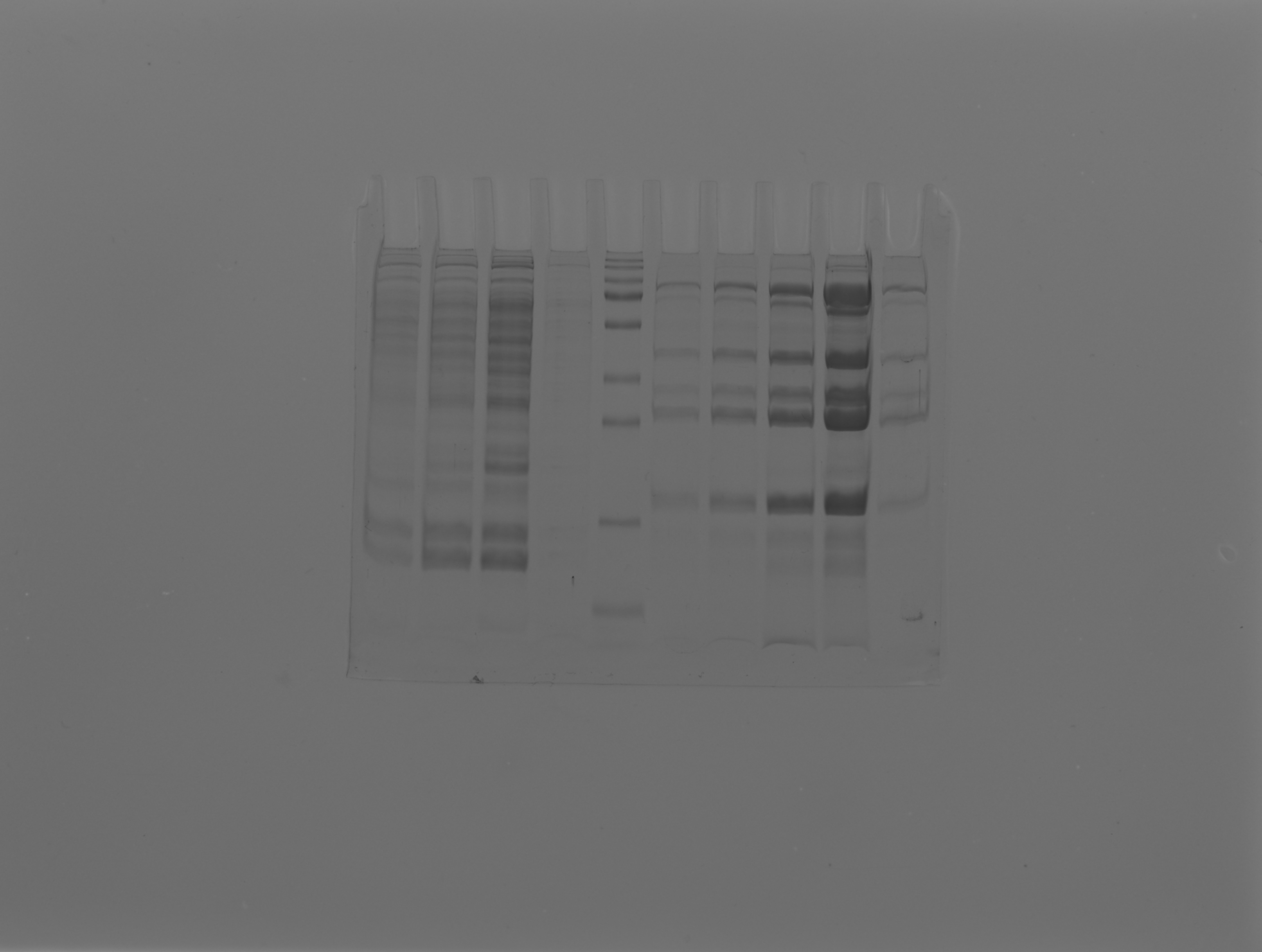

Supplement: Figure 2—source data 1. [file elife-81897-fig2-data1.zip › Figure 2-source data 1/Figure 2B_raw_uncropped.tif]

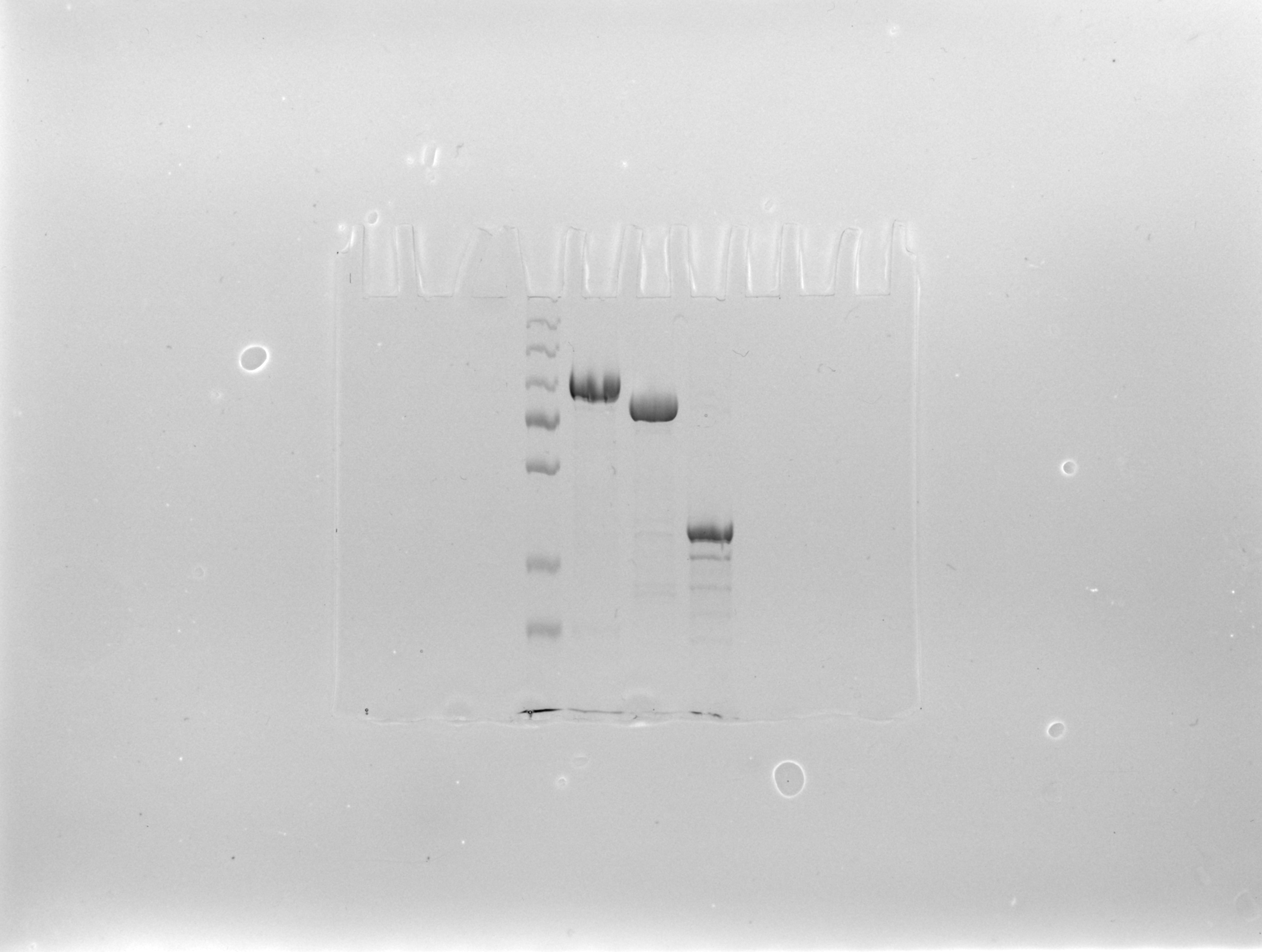

Supplement: Figure 2—source data 2. [file elife-81897-fig2-data2.zip › Figure 2-source data 2/Figure 2C_raw_uncropped.tif]

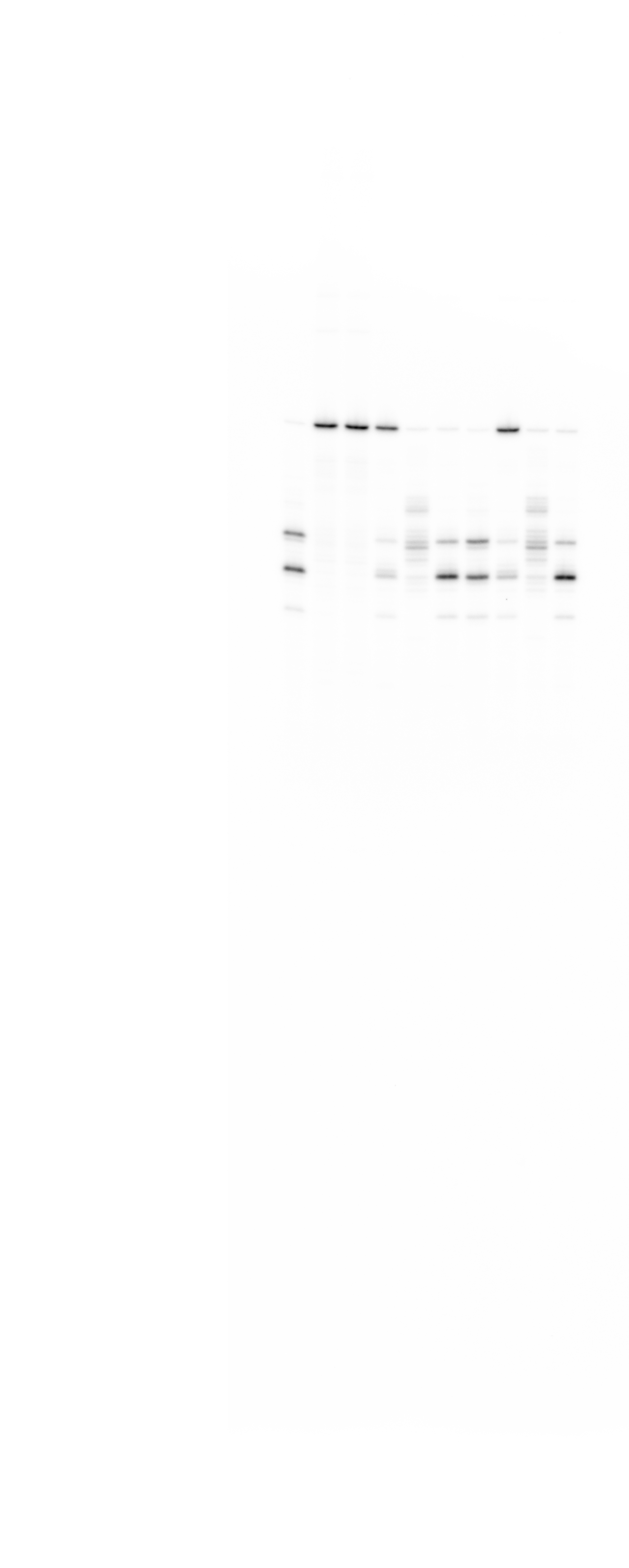

Supplement: Figure 2—source data 3. [file elife-81897-fig2-data3.zip › Figure 2-source data 3/Figure 2D_raw_uncropped.tif]

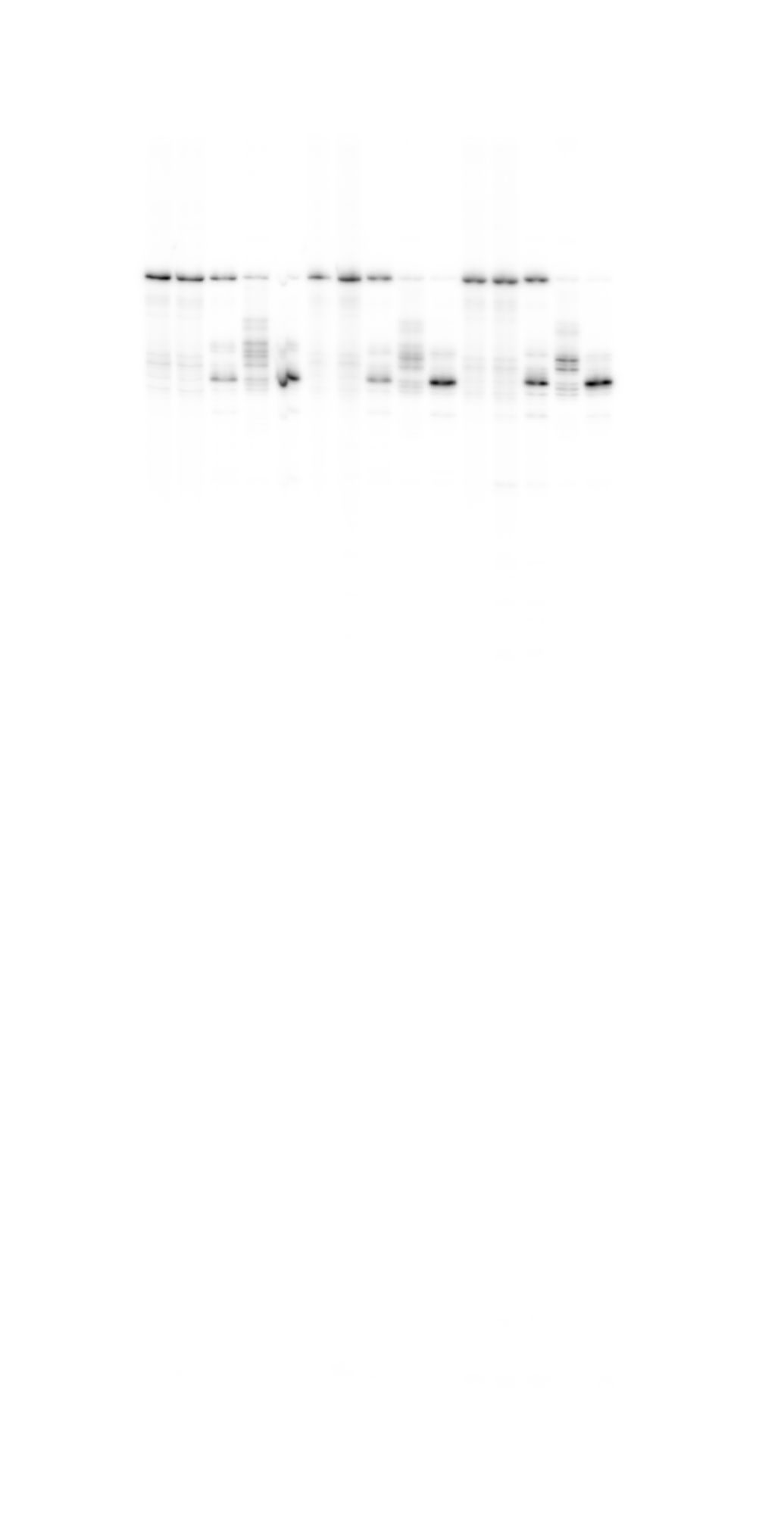

Supplement: Figure 2—figure supplement 1—source data 1. [file elife-81897-fig2-figsupp1-data1.zip › Figure 2-figure supplement 1-source data 1/Figure 2-figure supplement 2-raw_uncropped.tif]

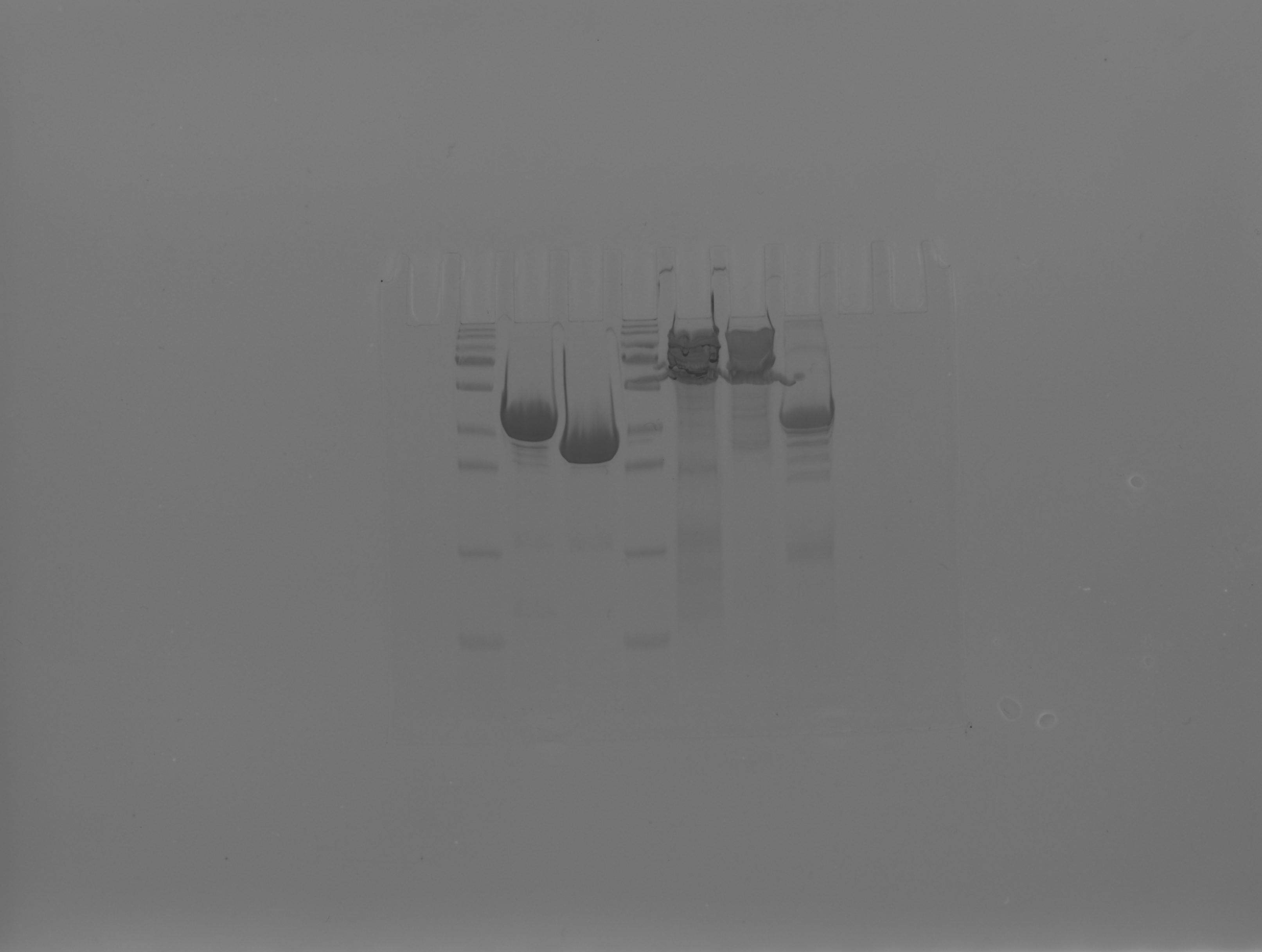

Supplement: Figure 3—source data 1. [file elife-81897-fig3-data1.zip › Figure 3-source data 1/Figure 3A_raw_uncropped.tif]

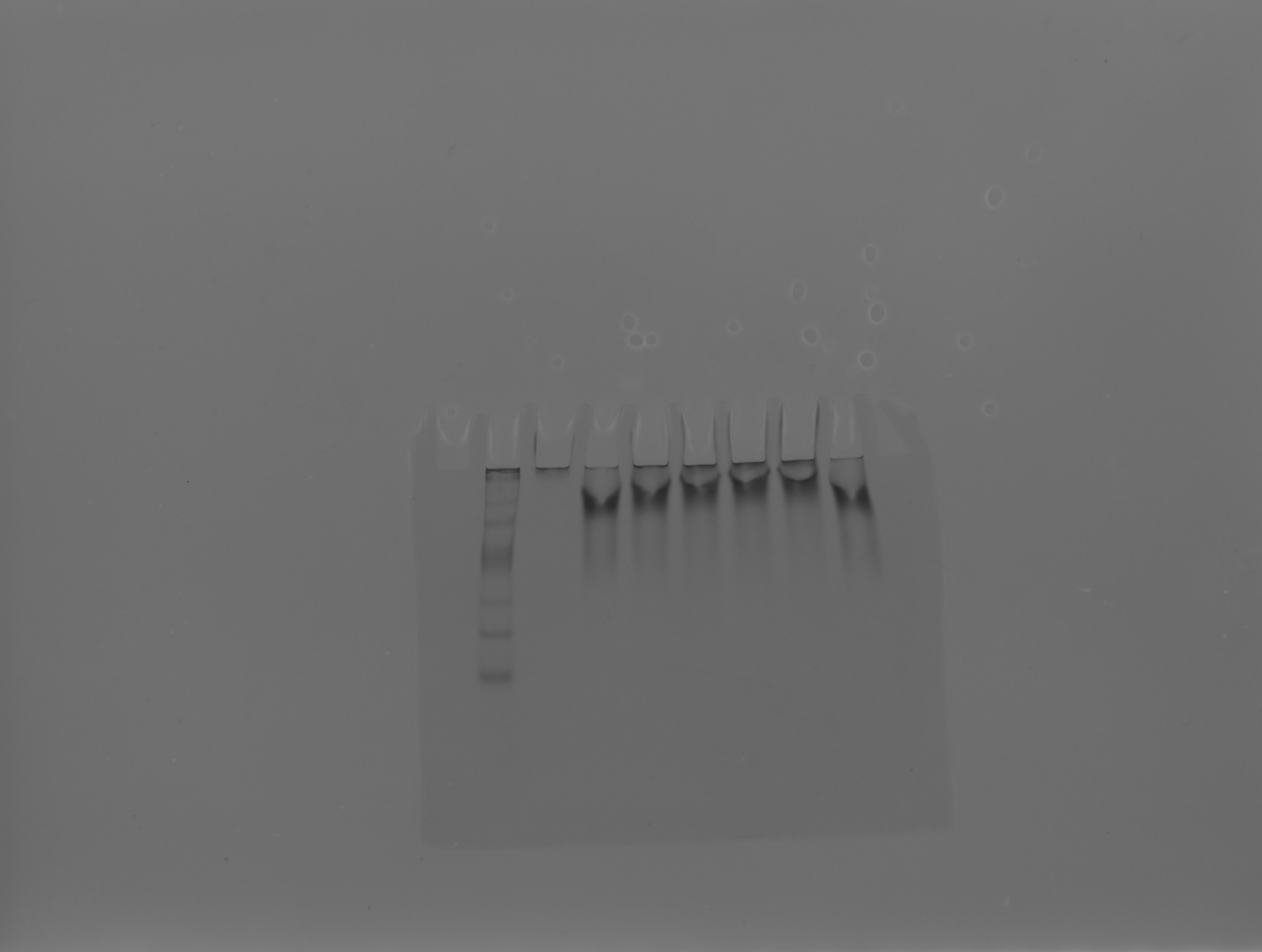

Supplement: Figure 3—source data 2. [file elife-81897-fig3-data2.zip › Figure 3-source data 2/Figure 3B_raw_uncropped.tif]

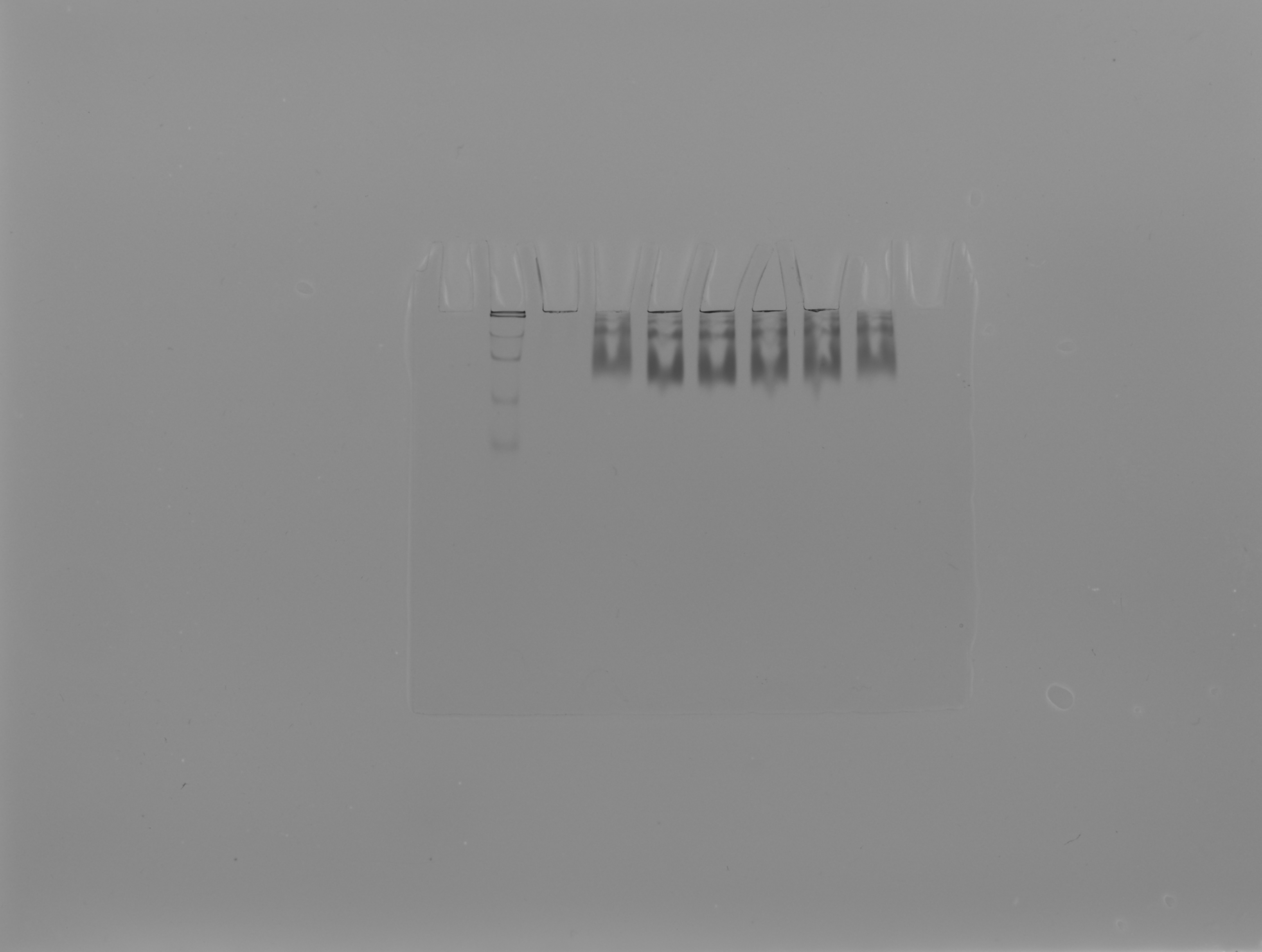

Supplement: Figure 3—figure supplement 1—source data 1. [file elife-81897-fig3-figsupp1-data1.zip › Figure 3-figure supplement 1-source data 1/Figure 3-figure supplement 1_raw_uncropped.tif]

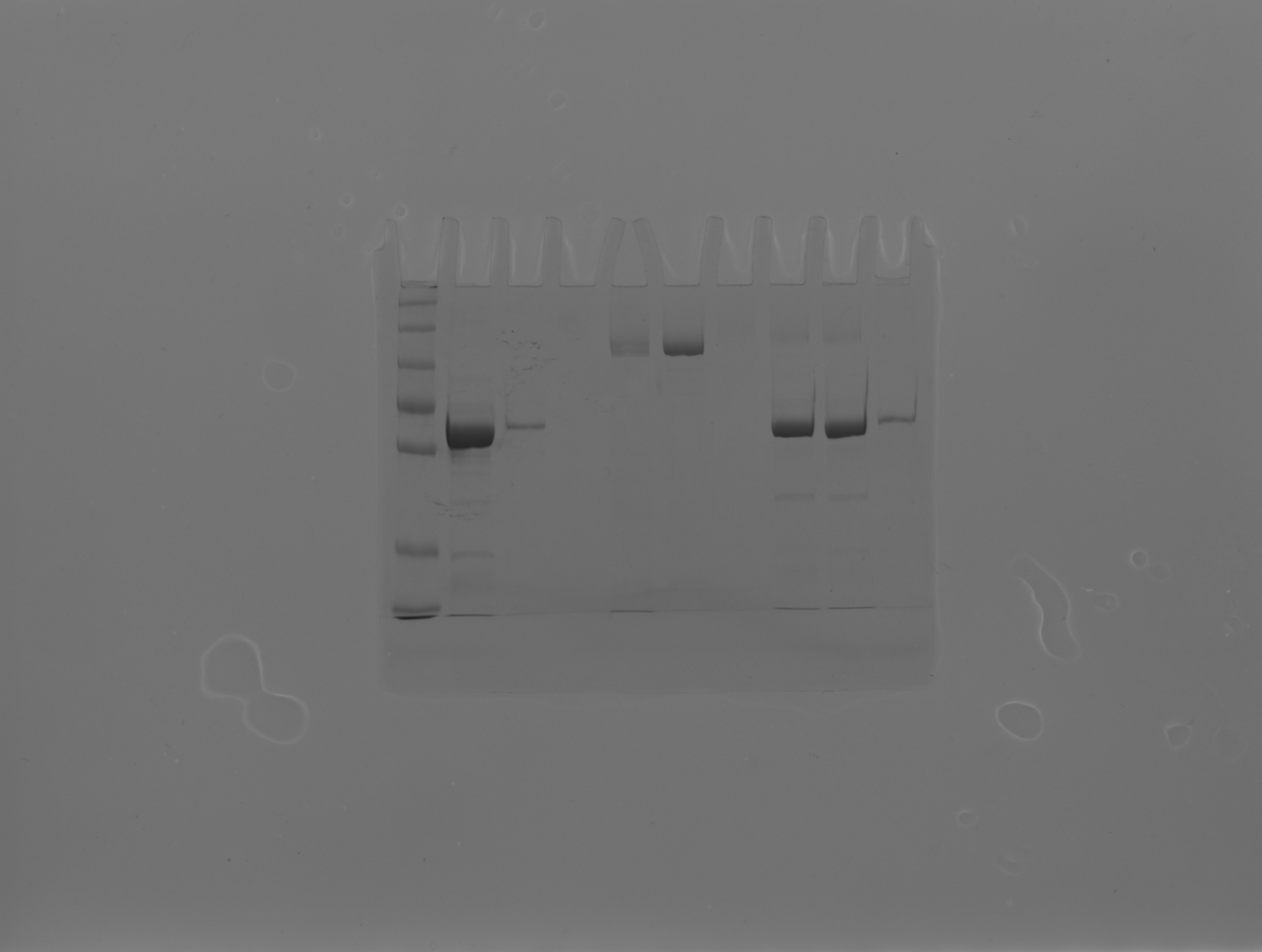

Supplement: Figure 3—figure supplement 2—source data 1. [file elife-81897-fig3-figsupp2-data1.zip › Figure 3-figure supplement 2-source data 1/Figure 3-figure supplement 2-panel B_raw_uncropped.tif]

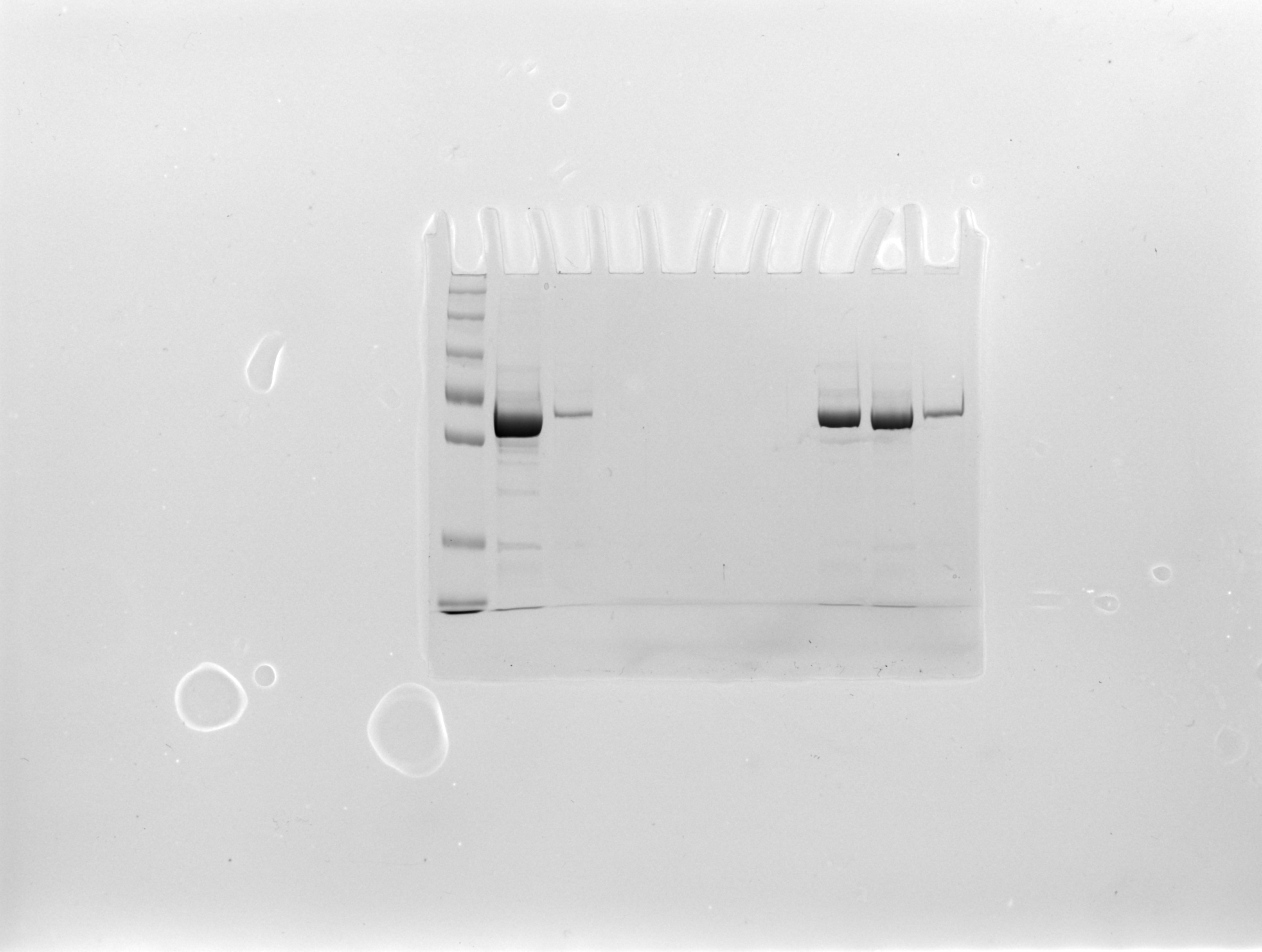

Supplement: Figure 3—figure supplement 2—source data 2. [file elife-81897-fig3-figsupp2-data2.zip › Figure 3-figure supplement 2-source data 2/Figure 3-figure supplement 2-panel C_raw_uncropped.tif]

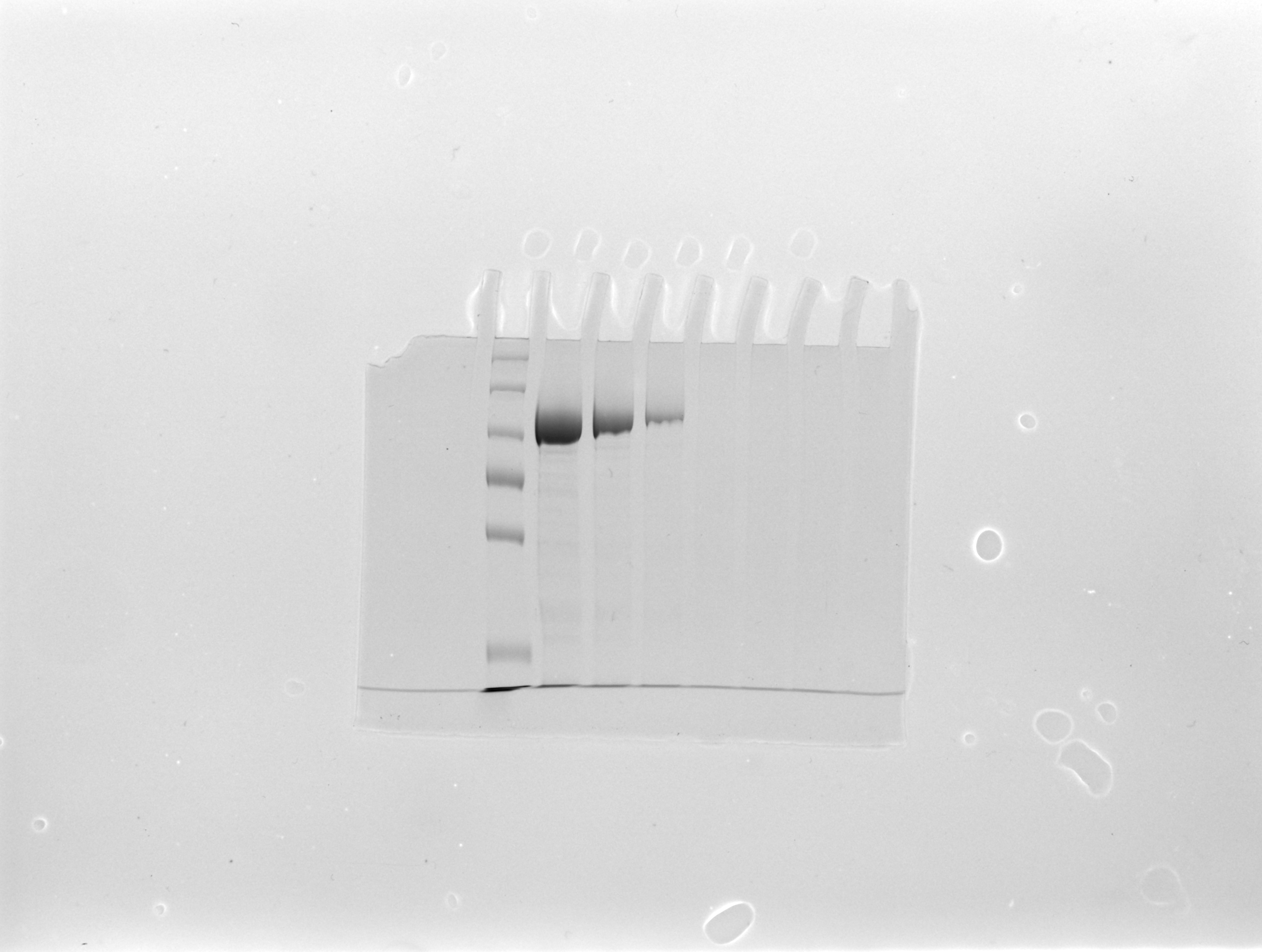

Supplement: Figure 3—figure supplement 2—source data 3. [file elife-81897-fig3-figsupp2-data3.zip › Figure 3-figure supplement 2-source data 3/Figure 3-figure supplement 2-panel D_raw_uncropped.tif]

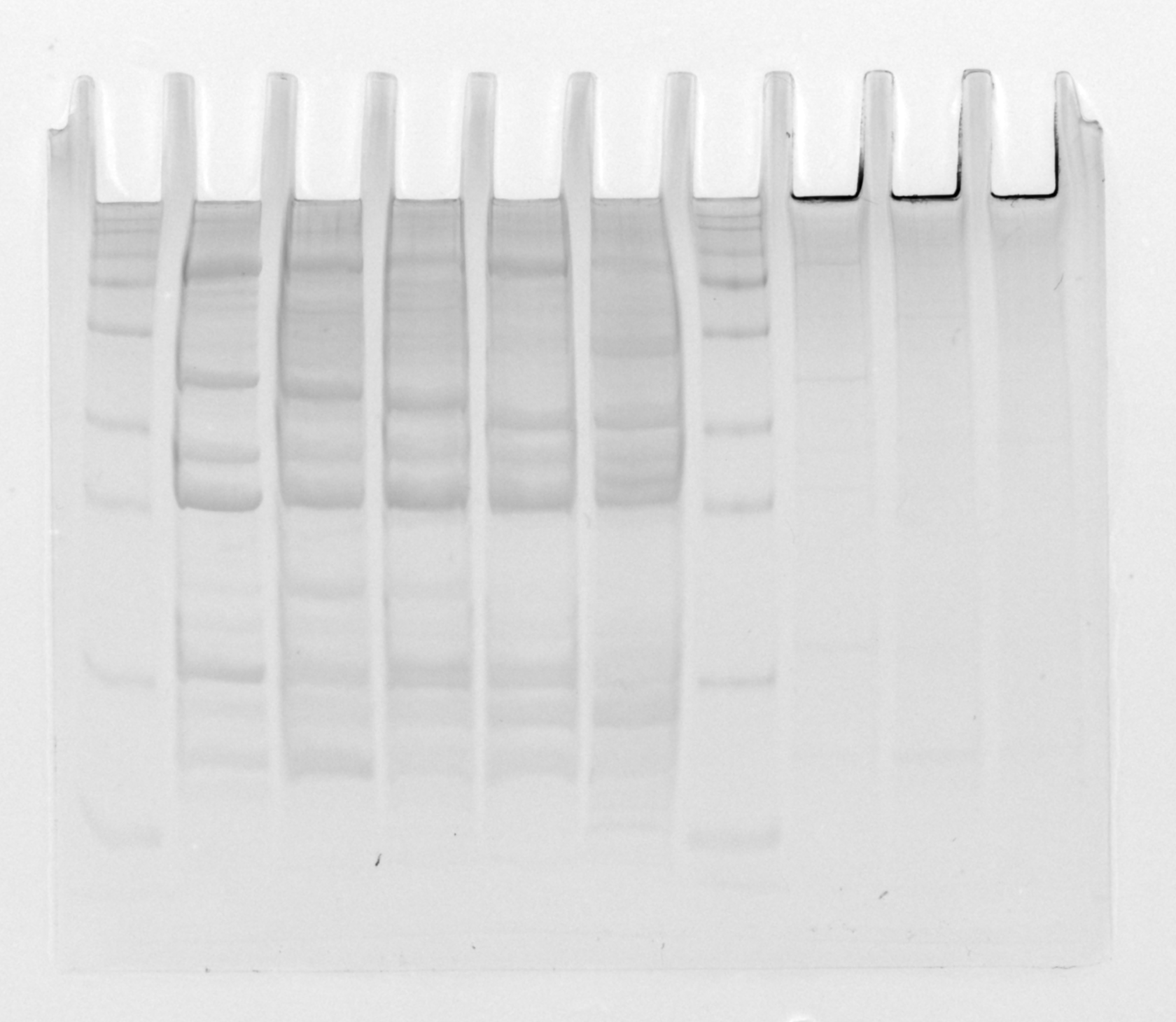

Supplement: Figure 4—source data 1. [file elife-81897-fig4-data1.zip › Figure 4-source data 1/Figure 4B_raw_uncropped.tif]

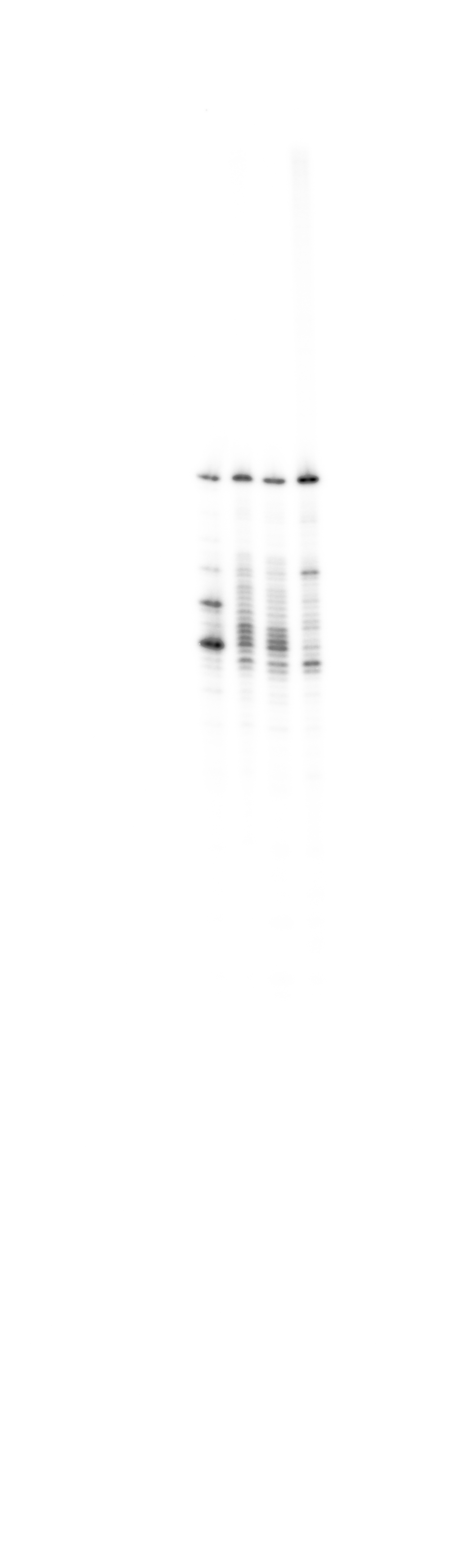

Supplement: Figure 4—source data 2. [file elife-81897-fig4-data2.zip › Figure 4-source data 2/Figure 4C_raw_uncropped.tif]

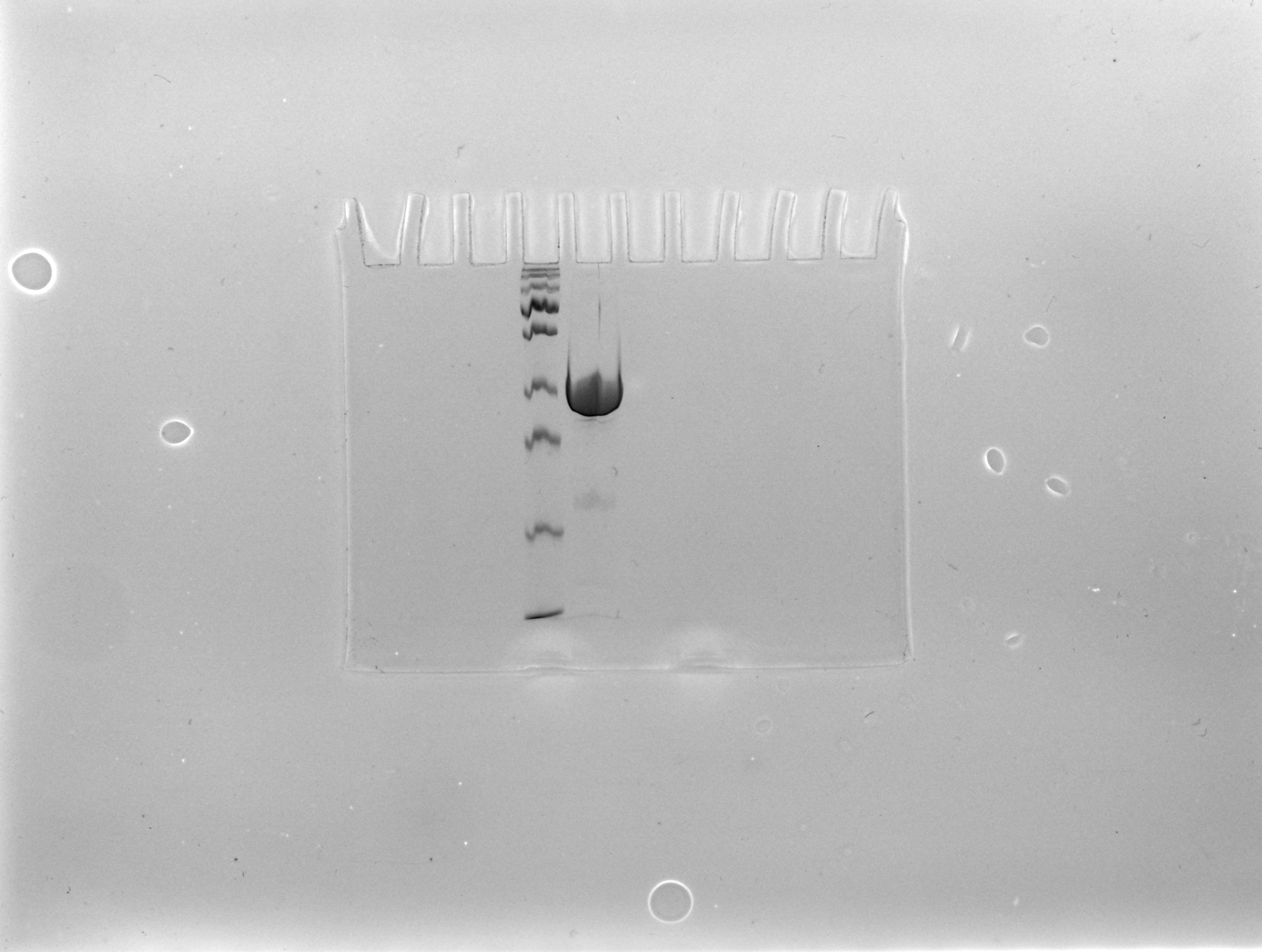

Supplement: Figure 5—source data 1. [file elife-81897-fig5-data1.zip › Figure 5-source data 1/Figure 5A_raw_uncropped.tif]

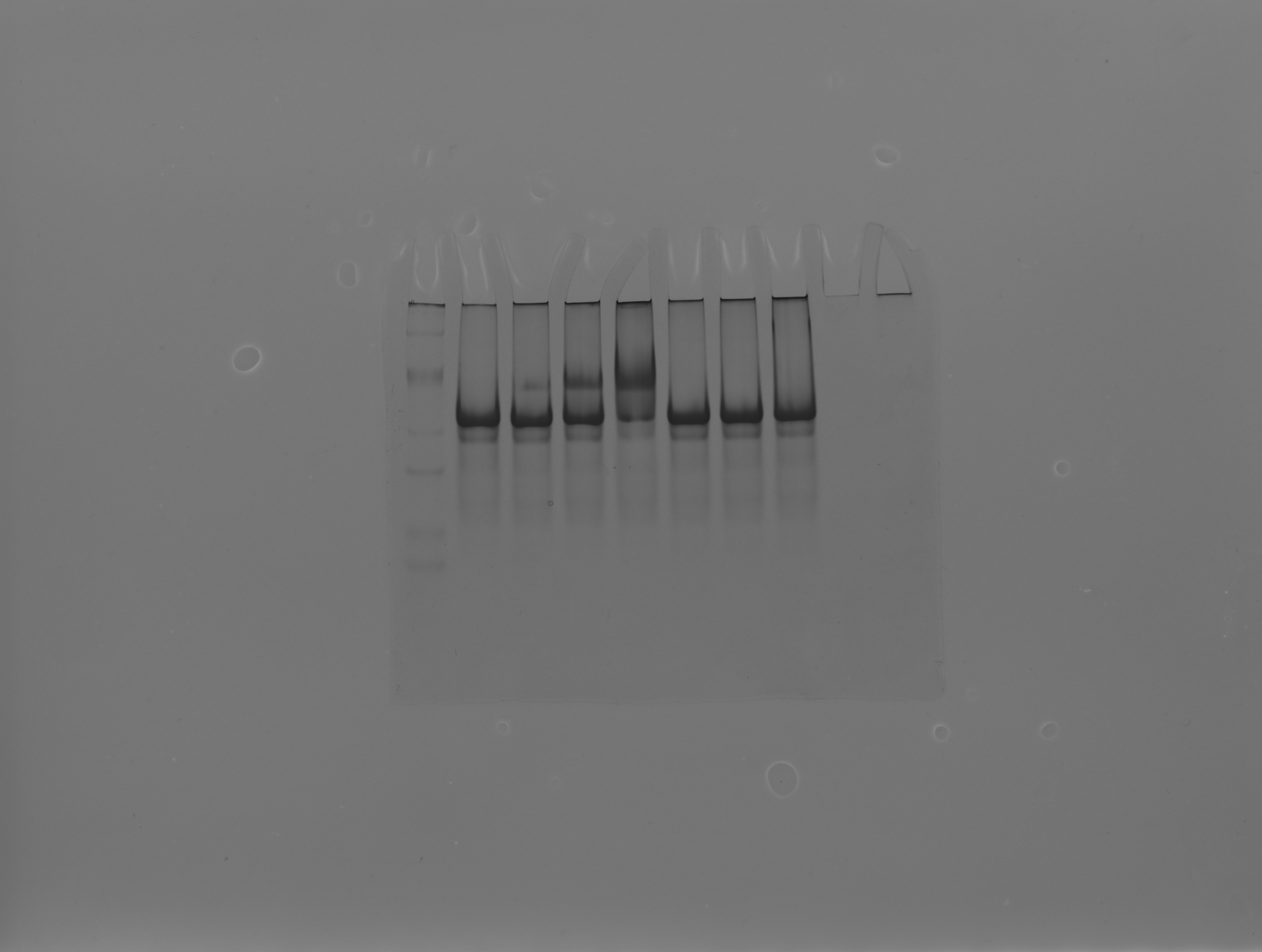

Supplement: Figure 5—source data 2. [file elife-81897-fig5-data2.zip › Figure 5-source data 2/Figure 5B_raw_uncropped.tif]

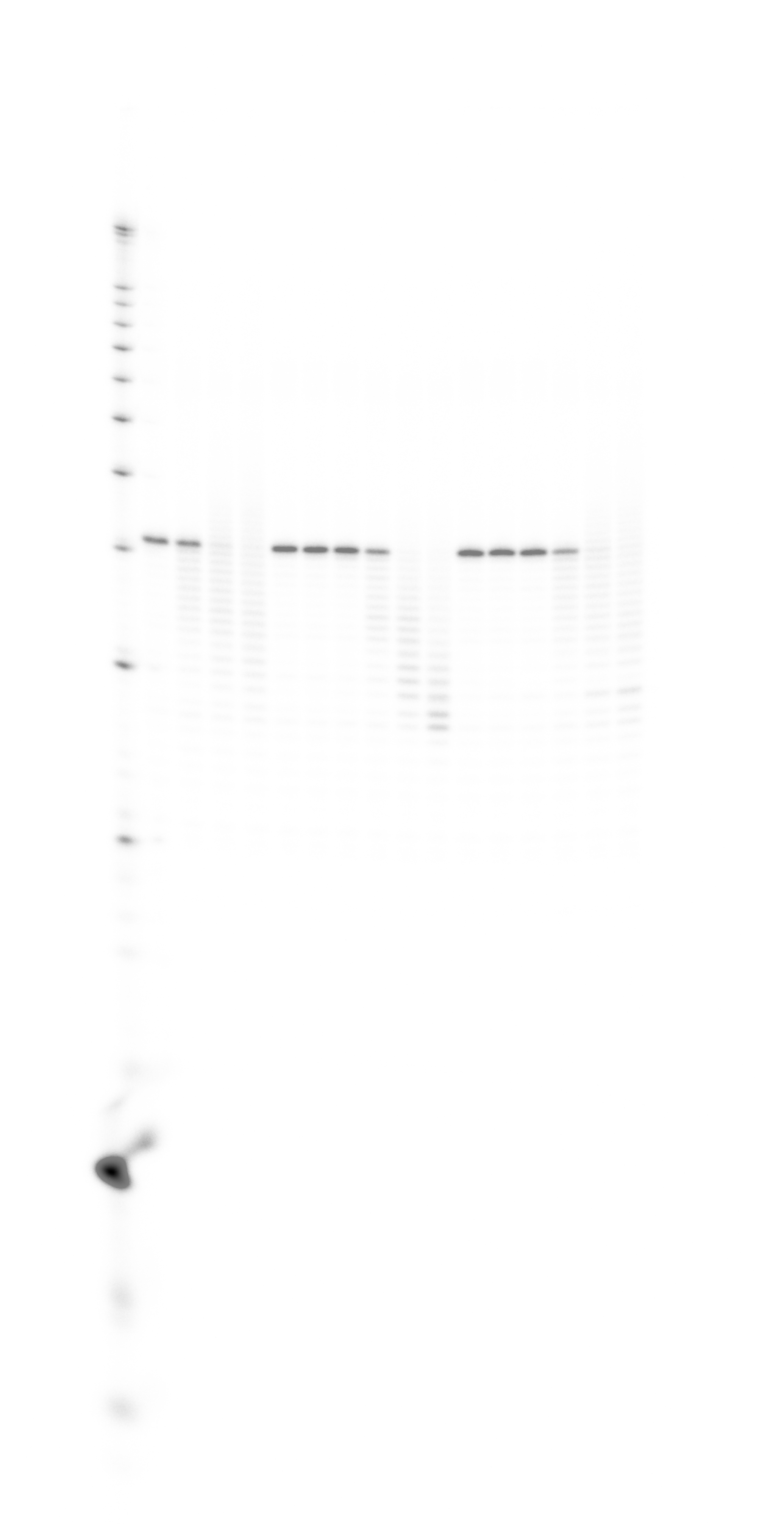

Supplement: Figure 5—source data 3. [file elife-81897-fig5-data3.zip › Figure 5-source data 3/Figure 5C_raw_uncropped.tif]

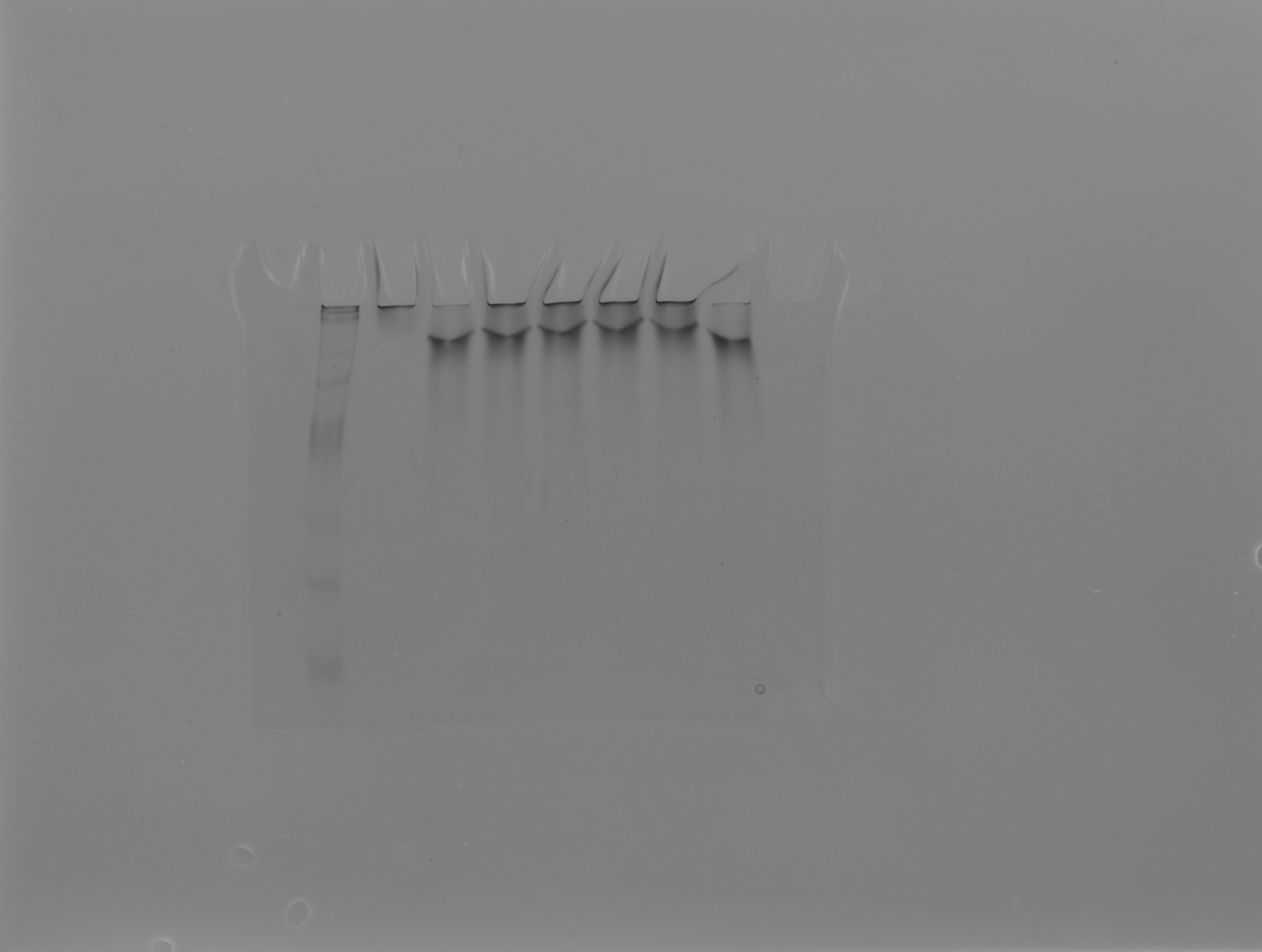

Supplement: Figure 5—figure supplement 1—source data 1. [file elife-81897-fig5-figsupp1-data1.zip › Figure 5-figure supplement 1-source data 1/Figure 5-figure supplement 1_raw_uncropped.tif]
