# Supplementary material for: Critical roles for ‘housekeeping’ nucleases in type III CRISPR-Cas immunity
Source: eLife. 2022 Dec 8;11:e81897. doi: 10.7554/eLife.81897 (PMC9762709; doi:10.7554/eLife.81897)
Supplement: Supplementary file 1. — Theoretical molecular weights and isoelectric points of purified proteins in this study. [file elife-81897-supp1.docx]

**Supplementary File 1.**  Accompanies Figures 2, 3, 5, Figure 3-figure supplement 1, and Figure 5-figure supplement 1. Theoretical molecular weights and isoelectric points of purified proteins in this study.

| **Protein** | **Molecular Weight (kDa)** | **Isoelectric Point** |
| --- | --- | --- |
| Csm5 WT | 39.3 | 9.51 |
| Csm5 Δ46 | 34.1 | 9.25 |
| Cbf1 | 35.7 | 6.46 |
| RNase R | 91.1 | 6.08 |
| PNPase | 77.4 | 4.92 |
| BSA | 69.3 | 5.82 |
