# Supplementary material for: Critical roles for ‘housekeeping’ nucleases in type III CRISPR-Cas immunity
Source: eLife. 2022 Dec 8;11:e81897. doi: 10.7554/eLife.81897 (PMC9762709; doi:10.7554/eLife.81897)
Supplement: Supplementary file 2. [file elife-81897-supp2.docx]

**Supplementary File 2.** DNA oligonucleotides used for cloning and PCR in this study.

| **Primer** | **Sequence (5’-3’)** | **Target** | **Purpose** |
| --- | --- | --- | --- |
| A481 | GCCATCCAGCTGATATCCCC | pKOR1 vector | Gibson Assembly:  pKOR1-Δ*rnr* |
| L138 | GGGGCCCGAGCTTAAGAC |  |  |
| L139 | GGATATCAGCTGGATGGCGACGATGTACTTGTCGGACTTTTTTATTTTATAATTG | Upstream arm from  *S. epidermidis* RP62a genome |  |
| L140 | CTTCTTCACCATTTCTTCGATGGATTGCTTTAAATTCATTATG |  |  |
| L141 | CATCGAAGAAATGGTGAAGAAGAAATCGCGTCGAAAG | Downstream arm from *S. epidermidis* RP62a genome |  |
| L142 | TCTTAAGCTCGGGCCCCCTATATAATGGAGACGGCGGGATT |  |  |
| L145 | CTCCAGATCCATATCCTTCTTTTTCTGAACC | pKOR1-based plasmids | To confirm insertion of upstream/downstream fragments in pKOR1-Δ*rnr* and pKOR1-*rnr** |
| L146 | GCTCGTATGTTGTGTGGAATTGTGAG |  |  |
| L143 | CAGCTGGATGGCACTAAAGACGGTATTACTGCGATTC | *S. epidermidis* RP62a genome | To confirm *rnr* knockout |
| L157 | TCATGTCTTGCTTTACGATTTTCAGC |  |  |
| L154 | GTAAATATTTAATAGAaAATTCaATGGATAAAAAAGAAATACGTC | *S. epidermidis* RP62a genome | To introduce silence mutations in *rnr* and gibson assembly: pKOR1-*rnr** |
| L155 | TTATCCATtGAATTtTCTATTAAATATTTACGAATTAATCTATGC |  |  |
| L153 | CAAATAGACAACTCGTATACTTTAAGTACAATATTAAGGAAG | *S. epidermidis* RP62a genome | To confirm *rnr** knock-in |
| F062 | TTATCTTTGATGATGCAATATATTAAGCAGCAAGAG | p*crispr-cas* | Gibson Assembly:  p*crispr-cas/csm5^H6N^*Δ18 |
| L246 | GAAAATGAAGGAAATATCATATCAGCAAGGAATGTGTAAAGTGAG |  |  |
| F063 | TTGCTGCTTAATATATTGCATCATCAAAGATAAACC |  |  |
| F066 | GCACCGAGATTATCTATATCGGCACGTACCACG |  |  |
| F067 | GGTACGTGCCGATATAGATAATCTCGGTGCTAC |  |  |
| L247 | CCTTGCTGATATGATATTTCCTTCATTTTCCCATAAGTTCCTCG |  |  |
| F061 | AATAGGCTAAACCATCTTAAATGTAGTATACTATTAATATAAATG | p*crispr-cas* | Gibson Assembly:  p*crispr-cas/csm5^H6N^*Δ31 |
| L265 | GCTGATATGAAGTTAAAATCTCAAAAGAATCTTGTTTAGCTTGTTTTC |  |  |
| L264 | CTTTTGAGATTTTAACTTCATATCAGCAAGGAATGTGTAAAGTGAG |  |  |
| F046 | ATATTAATAGTATACTACATTTAAGATGGTTTAGCCTATTC |  |  |
| L274 | CACACTATCAATTATCATATCAGCAAGGAATGTGTAAAGTGAG | p*crispr-cas* | Gibson Assembly:  p*crispr-cas/csm5^H6N^*Δ46 |
| L275 | CCTTGCTGATATGATAATTGATAGTGTGTTGTTTTACTAACAAATCCTG |  |  |
| A416 | TATTCTGAAAAGGTCAATCAAGG | p*crispr-cas*-based plasmids | To confirm *csm5* deletions |
| F113 | CTATCCTTAGTTGGCGTGCTCA |  |  |
| L253 | TCATATCAGCAAGGAATGTGTAAAGTGAG | *pET28b-H10Smt3-csm5* | Inverse PCR:  p*ET28b-H10Smt3-csm5Δ46* |
| L298 | TAATTGATAGTGTGTTGTTTTACTAACAAATCCTG |  |  |
| T7P | TAATACGACTCACTATAGGG | pET28b-based plasmids | To confirm *csm5* deletions |
| T7T | TATGCTAGTTATTGCTCAG |  |  |
| L196 | CACGTGATGTCTATAATTATTTGTTACCATCACTGGTTCTCGTCCCCTTTTCTTCGG | p*crispr-spcA1* | Inverse PCR: pc*rispr-spcA2* |
| L198 | GTTCACCTACATCGTGAATACTTTTCAACGTCTTGGTTCTCGTCCCCTTTTCTTCGG | p*crispr-spcA1* | Inverse PCR: pc*rispr-spcA3* |
| L277 | AAGAGTTTTATGAAGGTAATGATATTCTAGTAGATGTTCTCGTCCCCTTTTCTTCGG | p*crispr-spcA1* | Inverse PCR: p*crispr-spc-opt* |
| L278 | ATCTACTAGAATATCATTACCTTCATAAAACTCTTGTTCTCGTCCCCTTTTCTTCGG | p*crispr-spcA1* | Inverse PCR: p*crispr-spc-sub* |
| A200 | TTGTCAAAAAAAGTGACATATCATATAATCTTGTAC | p*crispr-spc*-based plasmids | To confirm insertion of designed spacers |
| F052 | CATTATTTGAACCAACAAACGACTTTTAGTATAACCAC |  |  |
